# [Retracted] PTEN enhances G2/M arrest in etoposide-treated MCF-7 cells through activation of the ATM pathway

**DOI:** 10.3892/or.2026.9127

**Published:** 2026-04-29

**Authors:** Ruopeng Zhang, Li Zhu, Lirong Zhang, Anli Xu, Zhengwei Li, Yijuan Xu, Pei He, Maoqing Wu, Fengxiang Wei, Chenhong Wang

Oncol Rep 35: 2707–2714, 2016; DOI: 10.3892/or.2016.4674

Subsequently to the publication of the above article, a concerned reader drew to the Editor's attention that the β-actin bands featured in Fig. 1A were also apparently included in the ‘Ctrl’ experiments for the phospho-P53 protein bands in Fig. 1B. In addition, a specific pair of P53 bands in Fig. 1B had also apparently been re-used as phospho-CDC25C bands in Fig. 6A, suggesting that these figures may have potentially undergone inappropriate editing.

After having evaluated these data independently in the Editorial Office, the concerns of the reader were shown to have been well-founded. Therefore, in view of the uncertainties regarding the assembly of some of the western blot data in Figs. 1 and 6, the Editor of *Oncology Reports* has decided that this paper should be retracted from the Journal on account of uncertainties with the presentation of the abovementioned data. The authors were asked for an explanation to account for these concerns, but the Editorial Office did not receive a reply. The Editor apologizes to the readership for any inconvenience caused.

